# Robson on the Morbid Adhesion of the Placenta, Etc.

**Published:** 1872-05

**Authors:** Robert Robson

**Affiliations:** Indiana


### ROBSON ON MORBID ADHESION OF THE PLACENTA, ETC.

BY ROBERT ROBSON, M. D., INDIANA.

On the 12th of October, 1871, I was requested to visit Mrs. B——, in Center Township, in this county, a lady of rather delicate habit of body, the mother of several children. Having several miles to ride, I found, on my arrival, that she had miscarried in the sixth month of gestation, the child being born one hour before my arrival. She stated that she had no premonitory symptoms, but was taken suddenly, and that the child was born without any pain whatever, and without any discharge of whatever character, that she was aware of; the child, upon inspection, appeared small and regularly developed, and gave no indication of the existence of disease in utero. On examination I found the uterus firmly contracted, presenting to the feel, a solid, hard mass, immediately above the symphisis pubis and per vagina. I could reach, with difficulty, the surface of a portion of the placenta, and felt convinced, from its high position in the fundal zone of the uterus, and entire exemption from hemorrhage, that it was wholly and firmly attached to that organ.

The funis, or cord, was broken high up, and was attached to the child one hour before I arrived; the vagina seemed small and contracted, and any attempt, on my part, to reach the placenta, was attended with pain, and rendered it impracticable to trace its connection, without introducing the hand, which, under the circumstances, I did not consider justifiable. Everything was done, by way of pressure, friction and the administration, from time to time, in proper doses, of the secale cornutum; but with no apparent effect. No bearing down pains could be produced. Here was, in my opinion, evidently a case of morbid adhesion of the placenta and uterus. After remaining several hours, the patient expressing herself free from pain, or suffering in any way, I ordered a dose of ol. recini, and left for home. On the following day I again made her a visit, and found her comparatively comfortable; no change in any way, having had no pain, nor the slightest sign of hemorrhage. The castor oil had operated well. Her pulse was natural and perfectly free from fever. I again left her, with special instruction to send for me immediately, should any uterine pains or flooding take place, but not hearing from her for several days, I naturally concluded that some other physician had been sent for, when to my surprise, five weeks from the first visit, I was again called, and on my arrival found that the placenta had been expelled, followed by the most alarming hemorrhage. The placenta was of a firm and healthy appearance, perfectly free from unpleasant fœtor, showing that general and vascular attachment necessary to carry on the circulation between the maternal and fœtal system. The hemorrhage was both alarming and uncontrolable, and was only relieved after the usual remedies, such as refrigerants, astringents, stimulants, friction, etc., had all failed, by the injection, per utero, of the per-chlorate of iron, frequently repeated.

*Remarks.*—Allow me to say that I have, of late, had repeated necessity to resort to this article, and I know of no medicine so reliable in uterine hemorrhage—none whose direct action in coagulating the blood in the mouths of the vessels, is so prompt and efficient; it provokes contraction of the muscular wall, and acts precisely, in those cases of powerless labor where there is no contractile energy to depend upon, rescuing the patient, as in the above instance, whose position would be otherwise hopeless. It is certainly an available power in obstetric practice.

